# Cross-Cultural Adaptation and Psychometric Evaluation of the Turkish Perceived Ageism Questionnaire (PAQ-TR)

**DOI:** 10.3390/healthcare13222844

**Published:** 2025-11-09

**Authors:** Mert Doğan, Özge Erol Doğan

**Affiliations:** 1 Department of Physiotherapy and Rehabilitation, Faculty of Health Sciences, Akdeniz University, Antalya 07070, Türkiye; mertdogan@akdeniz.edu.tr; 2 Department of Health Care Services, Vocational School of Health Services, Ardahan University, Ardahan 75000, Türkiye

**Keywords:** perceived ageism, psychometric properties, cross-cultural validation, reliability, older adults

## Abstract

Background/Objectives: Perceived ageism has been increasingly recognized as a critical determinant of the health and well-being of older adults. However, the cross-cultural validation of instruments measuring ageism remains limited. This study aimed to translate, culturally adapt, and evaluate the psychometric properties of the Turkish version of the Perceived Ageism Questionnaire (PAQ-TR). Methods: Content validity was assessed by a multidisciplinary expert panel. Construct validity was examined using exploratory (EFA) and confirmatory factor analyses (CFA). Convergent validity was tested through correlations with the Brief Aging Perceptions Questionnaire (B-APQ). Reliability was evaluated using Cronbach’s alpha, McDonald’s omega, and test–retest intraclass correlation coefficients (ICC). Statistical analyses were performed using IBM SPSS Statistics v27 (IBM Corp., Armonk, NY, USA) and AMOS v22 (IBM Corp., Chicago, IL, USA). Results: A total of 307 older adults (137 men and 170 women) participated in the study. The mean age was 71.19 (6.96) years, and 55.7% of the participants were female. An EFA confirmed the original two-factor structure, explaining 61.2% of the total variance, which was subsequently verified by a CFA, showing a good model fit. EFA confirmed the original two-factor structure, explaining 61.2% of the total variance (Kaiser-Meyer–Olkin = 0.82; Bartlett’s *χ^2^*(28) = 412.5, *p* < 0.001). The structure was subsequently verified by CFA, which demonstrated an excellent model fit (Comparative Fit Index = 0.97; Tucker–Lewis Index = 0.96; Root Mean Square Error of Approximation = 0.052; Standardized Root Mean Square Residual = 0.041). The Negative subscale showed high internal consistency (Cronbach’s alpha (*α*) = 0.84; McDonald’s Omega (*ω*) = 0.85), whereas the Positive subscale indicated moderate reliability (*α* = 0.58; *ω* = 0.60). The test–retest reliability was excellent, reflecting strong temporal stability (*Intraclass Correlation Coefficient* = 0.91). Convergent validity showed that the positive subscale was related to positive aging perceptions and less to negative ones, while the negative subscale showed the opposite pattern. Conclusions: The PAQ-TR demonstrated adequate psychometric properties for assessing perceived ageism among older adults in Türkiye. While the Negative subscale showed robust reliability, the positive subscale required refinement. These findings highlight both the cross-cultural validity of the PAQ and the influence of cultural norms on positive aging perception. The PAQ-TR is a valuable tool for research and clinical practice.

## 1. Introduction

Ageism—stereotypes, prejudices, and discrimination directed at individuals based on age—operates not only at the interpersonal level but also within institutional and structural domains, constituting a pervasive social determinant that undermines healthy aging and equity [1,2]. The World Health Organization’s Global Report on Ageism synthesizes the magnitude, mechanisms, and impacts of ageism and calls for strengthened measurement and monitoring to inform policy and intervention [1]. Quantitatively, a comprehensive systematic review spanning 422 studies across 45 countries (>7 million participants) demonstrated consistent associations between ageism and worse outcomes across 11 health domains, with effects evident at both the individual and structural levels [3]. For clinicians and health systems, these data frame ageism as a modifiable exposure with tangible implications for mental and physical health, social participation and quality of life [1,2,3].

Robust measurement is therefore indispensable for understanding ageism’s prevalence, tracking disparities, and evaluating anti-ageism strategies. Classic attitude measures—Kogan’s Attitudes Toward Old People [4], the Fraboni Scale of Ageism [5], and the Ambivalent Ageism Scale [6]—remain widely used; however, most were developed in Western contexts and require careful cross-cultural adaptation before use elsewhere to secure semantic and conceptual equivalence [7,8]. Addressing the subjective, day-to-day experience of ageism, the Perceived Ageism Questionnaire (PAQ-8) is a brief, two-factor instrument that differentiates negative and positive ageism and has shown solid psychometric performance in its original validation [9]. Since its development in Dutch samples (original validation), a Portuguese adaptation with psychometric evaluation has been published [10]. In China, the PAQ has been used as an outcome measure in community-based studies, although a formal, independent validation of the Chinese version has not been reported to date [11,12]. Collectively, this landscape underscores why cross-cultural adaptation and psychometric evaluation are preconditions for valid comparisons and clinical or public health applications across settings [7,8].

In Türkiye, there is a clear need for a concise, theory-consistent instrument with demonstrated reliability and validity to quantify perceived ageism in older adults and to support the surveillance, comparative research, and evaluation of practice and policy initiatives. Accordingly, the present study aimed to produce a Turkish cross-cultural adaptation of the PAQ (PAQ-TR) and examine its psychometric properties, including internal consistency, structural validity, and convergent/criterion validity, in community-dwelling older adults. By enabling a standardized assessment of perceived ageism, the PAQ-TR is expected to strengthen evidence-informed clinical practice and population health strategies in Türkiye [1,3,9].

## 2. Materials and Methods

### 2.1. Study Design and Reporting

This was a cross-sectional, methodological study that translated, cross-culturally adapted, and psychometrically evaluated the PAQ-TR. Reporting followed the STROBE recommendations for observational research and COnsensus-based Standards for the Selection of Health Measurement INstruments (COSMIN) guidance for studies on the measurement properties of patient-reported outcome measures [8,13].

### 2.2. Participants and Setting

Data were collected between March and July 2023 from community-dwelling older adults in Ardahan, Türkiye. Participants were recruited through posters, community announcements, and clinician referrals at a municipal senior center and the Department of Elderly Care at Ardahan University. The inclusion criteria were age ≥ 65 years, native-level Turkish proficiency, and the ability to complete self-report instruments independently. The exclusion criteria were as follows: self-reported conditions preventing questionnaire completion (e.g., severe uncorrected visual or hearing impairment), acute illness on the day of assessment, or cognitive/psychiatric conditions precluding informed consent.

According to psychometric literature and the COSMIN guidelines, it is recommended to include at least 5–10 participants per item and a minimum total sample of 200–300 participants to obtain stable factor solutions in exploratory and confirmatory factor analyses, as well as a minimum of 50 participants for test–retest reliability [8,14,15]. Accordingly, 307 community-dwelling older adults were included in this study, and to assess temporal stability, 74 participants completed the PAQ-TR twice, with a 7–14-day interval between administrations.

### 2.3. Outcome Measures

Demographic and health-related information were obtained using a structured questionnaire developed specifically for this study. The instrument captured the participants’ age, sex, height, weight, dominant hand, educational attainment, employment status, presence of chronic conditions, and regular medication use.

#### 2.3.1. Perceived Ageism Questionnaire (PAQ-8)

The PAQ-8 is an 8-item self-report measure capturing perceived ageism over the past year, comprising Negative Ageism (5 items) and Positive Ageism (3 items). The items were rated on a 5-point frequency scale. Subscale scores were calculated as item means (higher scores reflected more frequent experiences of the corresponding construct). Consistent with the original study, the analyses focused on the subscales [9]. Permission to translate and use the instrument was obtained from its developers.

#### 2.3.2. The Brief Aging Perceptions Questionnaire (B-APQ)

The B-APQ was administered to assess convergent validity. The B-APQ is a multidimensional instrument developed to evaluate individuals’ cognitive representations of aging. It includes five subscales: timeline–chronic, positive consequences, positive control, negative consequences and control, and emotional representations. Items were rated on a five-point Likert-type scale ranging from strong disagreement to strong agreement. Higher scores reflect a stronger endorsement of the respective construct [16,17]. The B-APQ has been adapted and validated for use in older Turkish populations and has demonstrated satisfactory psychometric properties in this context [18].

### 2.4. Translation and Cross-Cultural Adaptation

The translation and cultural adaptation of the PAQ-8 from its original English version into Turkish (PAQ-TR) were carried out in accordance with the COSMIN guidelines [8,15]. All stages of adaptation were performed following COSMIN’s rigorous methodological framework to ensure semantic, idiomatic, experiential, and conceptual equivalences between the source and target versions. The comprehensive process included the following steps:

1. Forward Translation: Two independent bilingual translators produced two distinct Turkish versions (T1 and T2) of the original instrument. The translators had different backgrounds: one was a subject-matter expert health professional (to ensure clinical relevance), and the other was a professional linguistic translator (to ensure idiomatic precision).
2. Reconciliation: The two forward translations (T1 and T2) were meticulously compared and reconciled during a structured consensus meeting. The meeting was moderated by a bilingual researcher who synthesized the versions to resolve discrepancies and create a unified, reconciled Turkish version.
3. Back-Translation: Two native English speakers, who were blinded to the original questionnaire, independently back-translated the reconciled Turkish version back into English. This crucial step aimed to verify conceptual consistency and ensure that the meaning of the items was preserved in Turkish.
4. Expert Committee Review: A multidisciplinary panel reviewed all existing versions (original, forward translations, reconciled version, and back translations) to ensure both cross-cultural equivalence and content accuracy.
5. Cognitive Debriefing (Pilot Testing) The pre-final Turkish version was pilot-tested with a sample of 10 community-dwelling older adults from the target population. This cognitive debriefing aimed to evaluate the clarity, comprehensibility, and cultural relevance of the instrument. Based on the feedback received, minor wording and structural adjustments were made to the instrument before final approval.

During the cross-cultural adaptation of the PAQ-8 into Turkish, several precise linguistic and structural modifications were made to ensure conceptual fidelity while achieving linguistic and cultural naturalness. All items were rephrased into a uniform interrogative structure beginning with “*ne sıklıkta*” (“how often”) and ending with “*… hissine kapıldınız?*” (“have you had the feeling that…”) to standardize syntax, enhance readability, and align with the Turkish grammatical and cognitive style of self-report questioning.

In Items 1, 2, 4, 6, and 7, the original declarative forms (“people approach you…”, “you are not being listened to…”, “people hold negative prejudices…”, etc.) were converted into perception-based experiential phrasing (“*insanların size … hissine kapıldınız mı?*”), thereby preserving the construct of perceived rather than objective ageism. Lexical substitutions were also made to improve idiomatic accuracy: “sensible” was translated as “*mantıklı*” instead of “*aklı başında*”; “disparagingly” as “*aşağılayıcı*” rather than a literal “*küçümseyici biçimde*”; and “less capable” as “*daha az becerikliymişsiniz*”, which conveys both cognitive and functional connotations relevant in Turkish.

Item 8 underwent the most substantial cultural adjustment. The original examples referring to “babysitting (grand) children or volunteer activities” were expanded to include culturally typical unpaid support roles—“*bebek veya çocuk (torun) bakıcılığı ya da gönüllü faaliyetler (örneğin birilerine destek olmak amacıyla para kazanmadan yapılan temizlik, tamirat ve tadilat işleri)*”—to reflect the informal caregiving and household assistance commonly performed by older adults in Türkiye. Minor lexical harmonization was applied across all items (e.g., consistent *use of “yaşınız nedeniyle” for “because of your age”*) *to* ensure terminological consistency. The 5-point Likert response scale was retained, without modification (Appendix A.2).

### 2.5. Statistical Analysis

Statistical analyses were performed using IBM SPSS Statistics v27 (IBM Corp., Armonk, NY, USA) and AMOS v22 (IBM Corp., Chicago, IL, USA). A two-tailed significance level of 0.05 was applied to all the analyses.

The data quality and distributional properties were examined prior to the main analyses. Descriptive statistics, including means, standard deviations, medians, and minimum–maximum values, were computed for each item. Normality assumptions were tested using the Kolmogorov–Smirnov and Shapiro–Wilk tests, supported by visual inspection of histograms and Q–Q plots as well as skewness and kurtosis coefficients

#### 2.5.1. Content Validity

Content validity was assessed by a multidisciplinary expert panel consisting of ten professionals: two internal medicine specialists, two nurses, one gerontologist, two dietitians, and three physiotherapists. Each expert independently evaluated the relevance, conceptual coverage, and clarity of each item using a four-point ordinal scale ranging from “1 = not relevant” to “4 = highly relevant.” From these ratings, the item-level content validity index (I-CVI), average scale-level content validity index (S-CVI/Ave), and modified kappa (κ*) were calculated. An I-CVI ≥ 0.78 and S-CVI/Ave ≥ 0.80 were considered acceptable, while values ≥ 0.90 were interpreted as indicating excellent content validity [19,20].

#### 2.5.2. Structural Validity

Structural validity was investigated using exploratory factor analysis (EFA) and confirmatory factor analysis (CFA). First, an EFA was conducted to explore the underlying factor structure of the Turkish version. The extraction method was Principal Axis Factoring with Oblimin rotation, allowing for correlations between factors. Sampling adequacy and sphericity were verified using the Kaiser–Meyer–Olkin (KMO) measure and Bartlett’s test of sphericity. Factors with eigenvalues greater than 1.0 and item loadings of ≥0.30 were considered acceptable. A two-factor model was tested using AMOS v22, and multiple indices of global model fit were reported, including the chi-square test, chi-square/df ratio, the Comparative Fit Index (CFI), the Tucker–Lewis Index (TLI), the Root Mean Square Error of Approximation (RMSEA) with 90% confidence interval, and the Standardized Root Mean Square Residual (SRMR). Based on established guidelines, CFI and TLI values ≥ 0.95, RMSEA ≤ 0.06, and SRMR ≤ 0.08 were considered indicative of a good model fit. Standardized factor loadings ≥0.30 were regarded as salient [21]. Given the ordinal nature of the 5-point Likert items, subscale scores were treated as continuous, which is acceptable for ≥5-point scales in psychometric validation. The model was estimated in AMOS using maximum likelihood (ML) and verified with an ordinal estimator (WLSMV with polychoric correlations); both yielded consistent results.

#### 2.5.3. Convergent Validity

Convergent validity was examined through hypothesis testing, a standard approach in psychometric validation that involves pre-defining expected correlations with theoretically related measures [15,22]. It was hypothesized a priori that the Positive subscale of the PAQ-TR would correlate positively (r ≈ 0.20–0.35) with the Positive Consequences and Positive Control subscales of the B-APQ, and negatively (r ≈ −0.20 to −0.35) with the Negative Consequences/Control and Emotional Representations subscales of the B-APQ. Conversely, the Negative subscale of the PAQ-TR was expected to correlate positively (r ≈ 0.20–0.35) with the negative B-APQ domains (Negative Consequences/Control, Emotional Representations) and negatively (r ≈ −0.20 to −0.35) with the positive domains (Positive Consequences, Positive Control). These directional hypotheses align with established construct validity principles, whereby measures of theoretically similar constructs should demonstrate positive correlations, whereas measures of opposing or unrelated constructs should show negative or weak correlations [23]. Pearson’s and Spearman’s correlation coefficients were computed to test these hypotheses, consistent with the recommended practices for assessing convergent validity in scale validation studies [22,24]. To control for multiple testing across the seven B-APQ domains, the Benjamini–Hochberg False Discovery Rate (FDR) correction was applied to the correlation *p*-values. In this procedure, *p*-values were ranked in ascending order, and for each test i, the adjusted value was computed as

q₍ᵢ₎=( p₍ᵢ₎×m )/i

where *m* is the total number of tests conducted. A sequential (monotonic) adjustment was then applied to ensure that the *q*-values were non-decreasing. Associations were considered statistically significant if *q* < 0.05, and meaningful if they were both significant after FDR correction and of at least small-to-moderate magnitude (r ≥ 0.20).

#### 2.5.4. Reliability

Internal consistency was evaluated using Cronbach’s alpha (α) and McDonald’s omega (ω) coefficients. Test–retest reliability was assessed using a two-way mixed-effects model with absolute agreement, computing the intraclass correlation coefficient (ICC (3,1)) and 95% confidence intervals. Reliability was interpreted as poor (<0.50), moderate (0.50–0.75), good (0.75–0.90), or excellent (>0.90) [25]. The standard error of measurement (SEM) and smallest detectable change at 95% confidence (SDC_95_ = 1.96 × √2 × SEM) were calculated to facilitate interpretation at the individual level [25]. The agreement between the test and retest scores was further examined using Bland–Altman analysis, in which mean differences and 95% limits of agreement (mean ± 1.96 × SD) were calculated to assess potential systematic bias or proportional trends between administrations [26].

## 3. Results

### 3.1. Participant Characteristics

A total of 307 older adults participated in this study. The mean age was 71.19 years (SD = 6.96), and 55.7% of the participants were female. The mean height and weight were 165.92 cm (SD = 9.33) and 75.05 kg (SD = 14.87), respectively. Most of the participants were right-hand dominant (89.9%), not currently employed (98.7%), and had a primary school education (73.6%). In addition, 58.8% of the participants reported having at least one chronic disease, and 58.5% reported regular medication use (Table 1).

The descriptive results for the Turkish version of the PAQ-TR and B-APQ are presented in Table 2.

The mean (SD) scores for the PAQ-TR subscales were 10.66 (2.62) for Positive, 10.48 (3.87) for Negative, and 21.14 (3.64) for the total score. For the B-APQ, the highest mean was observed in the negative consequences/control domain (16.92 ± 4.91), whereas the emotional representation domain showed the lowest mean score (6.89 ± 1.89).

### 3.2. Content Validity

The content validity indices (I-CVI and S-CVI) calculated from the cumulative expert ratings for each questionnaire item are summarized in Table 3.

The I-CVI values ranged from 0.83 to 1.00. The S-CVIs for relevance, clarity, and comprehensiveness were 0.95, 0.90, and 0.86, respectively, with an overall mean S-CVI of 0.93, indicating satisfactory content validity. The modified kappa (κ*) coefficients ranged from 0.82 to 1.00, indicating excellent agreement among the experts.

### 3.3. Confirmatory Factor Analysis

The pre-analysis results supported the suitability of the data for factor analysis. Before the CFA, an EFA was conducted to examine the latent structure of the Turkish version. Using principal axis factoring with oblimin rotation, two factors with eigenvalues >1.0 emerged, jointly explaining 61.2% of the total variance. Sampling adequacy was verified (KMO = 0.82; Bartlett’s Test of Sphericity *χ^2^*(28) = 412.5, *p* < 0.001).

Factor 1 consisted of items 1, 2, 4, 6, and 7, reflecting negative ageism, whereas Factor 2 comprised items 3, 5, and 8, reflecting positive ageism. All salient loadings exceeded 0.30, and no cross-loadings exceeded 0.25. This two-factor solution was fully consistent with the theoretical model of the original PAQ-8, providing empirical support for proceeding to CFA.

**Figure 1:**
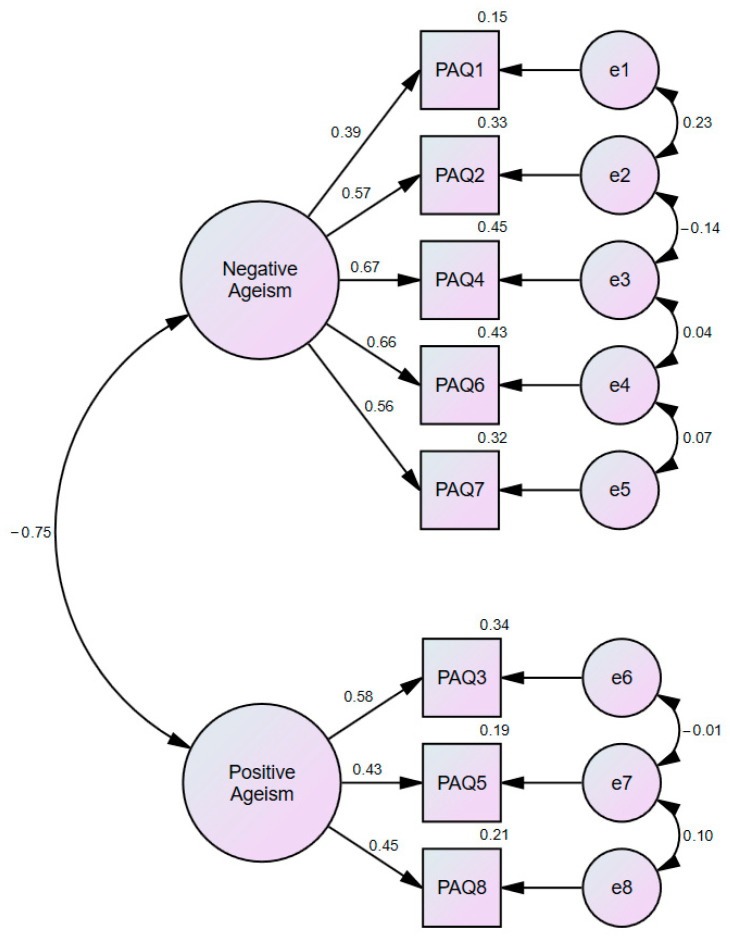
Confirmatory Factor Analysis Model of PAQ-TR.

The hypothesized two-factor structure of the PAQ-TR (Positive and Negative) demonstrated an acceptable to excellent model fit (Figure 1, Table 4).

Standardized factor loadings ranged from 0.39 to 0.67 for the negative factor and from 0.43 to 0.58 for the positive factor, with explained variances between 15.0% and 45.2% and 18.6% and 33.6%, respectively (Table 5).

Although composite reliability was adequate for the negative factor, it was lower for the positive factor. Similarly, the average variance extracted values were below the recommended 0.50 threshold (Negative = 0.34; Positive = 0.24). Despite these limitations, the moderate correlation between the latent factors (r = −0.45), compliance with the Fornell–Larcker criterion, and a satisfactory HTMT ratio (0.76 < 0.85) jointly support the adequacy of discriminant validity within the two-factor PAQ-TR measurement model [27].

### 3.4. Convergent Validity

The correlations between the PAQ-TR and B-APQ subscales are shown in Table 6.

As hypothesized, positive ageism was positively correlated with the adaptive domains of the B-APQ (positive consequences: r = 0.27; positive control: r = 0.20) and negatively correlated with maladaptive domains (negative consequences/control: r = −0.15; emotional representations: r = −0.12). Conversely, negative ageism showed significant negative correlations with adaptive domains (r = −0.29 and r = −0.23) and positive correlations with maladaptive domains (r = 0.31 and r = 0.25, respectively). These results support the convergent and criterion-related validity of the PAQ-TR.

### 3.5. Reliability

The internal consistency indices are presented in Table 7.

Cronbach’s alpha and McDonald’s omega coefficients indicated acceptable reliability for the PAQ-Negative subscale (*α* = 0.72, *ω* = 0.71) and total score (*α* = 0.74). The PAQ-positive subscale demonstrated lower reliability (*α* = 0.51, *ω* = 0.48), which may be attributed to its shorter item length. The mean inter-item correlations (MIIC) supported these findings, with values of 0.34 for the negative factor and 0.26 for the positive factor.

The test–retest reliability results are presented in Table 8.

Agreement was good for both subscales, with intraclass correlation coefficients of 0.80 for positive ageism and 0.86 for negative ageism, respectively. The standard errors of measurement (SEM) values were 1.16 and 1.44, and the smallest detectable change at the 95% confidence level (SDC_95_) was 3.22 and 3.99, respectively. Item-level stability ranged from 0.68 to 0.82, further supporting PAQ-TR’s temporal reliability.

**Figure 2:**
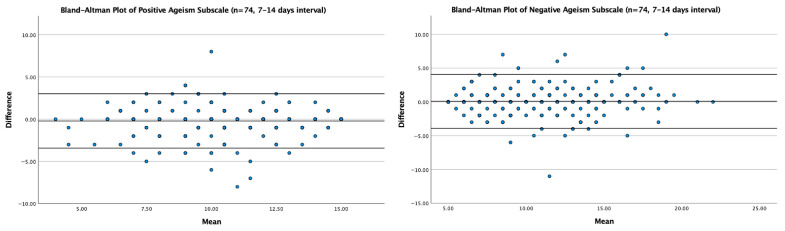
Bland–Altman plots illustrating the agreement between test and retest scores for the positive (**left**) and negative (**right**) ageism subscales (n = 74; 7–14 days interval). Solid lines represent mean differences (bias); dashed lines represent 95% limits of agreement.

The agreement between the test and retest scores was further examined using the Bland–Altman analysis (Figure 2). For the Positive Ageism subscale, the mean difference between the test and retest scores was 0.08 (SD = 2.03), with 95% limits of agreement ranging from −3.90 to +4.06. For the Negative Ageism subscale, the mean difference was −0.21 (SD = 1.64), with 95% limits of agreement from −3.43 to +3.01. Most data points fell within these limits, demonstrating good test–retest agreement and indicating no systematic bias or proportional trends across the measurement range.

## 4. Discussion

The findings of this study indicate that the Turkish version of the PAQ (PAQ-TR) demonstrates generally adequate psychometric properties for assessing perceived ageism among older adults in Türkiye. The prerequisites for factor analysis were met, and the fit indices obtained from the confirmatory factor analysis were within acceptable thresholds. The two-factor structure was confirmed, with the negative subscale showing strong reliability, while the positive subscale displayed more limited consistency. Furthermore, the test–retest results confirmed the temporal stability of the subscales, and the measurement error and smallest detectable change values enhanced the interpretability of the instrument for practical use. Convergent validity analyses were largely consistent with the pre-specified hypotheses.

These findings are consistent with the original PAQ-8 study. Brinkhof and colleagues also reported that the scale showed a two-factor structure in its development study, with the negative and positive dimensions of perceived ageism being distinct and structural validity confirmed [9]. Similarly, a recent validation study of the Portuguese PAQ-8 found that the two-factor structure was retained, Cronbach’s alpha coefficients were within acceptable levels, and particularly that the negative subscale demonstrated strong internal consistency. The Portuguese sample also revealed that both younger and older age groups reported higher levels of perceived ageism than middle-aged adults and that men scored higher than women on the negative subscale [10]. Thus, the heterogeneity of the positive dimension may not be unique to Türkiye but rather reflects a structural challenge inherent to this subscale. Indeed, a systematic review of ageism measures concluded that many existing instruments still lack robust psychometric evidence [28].

In the Turkish cultural context, our findings demonstrate the coexistence of both positive and negative attributions toward older adults. The prominence of negative perceptions may reflect older individuals’ frequent exposure to stereotypes, discrimination, or patronizing attitudes. In contrast, the weaker and more heterogeneous performance of the positive subscale may be explained by the fact that attributes such as “respect” and “wisdom” are regarded by some participants as cultural values, but by others as less salient in everyday experiences. At this point, reference to the concept of “benevolent ageism” is particularly relevant: Benevolent ageism describes superficially positive attitudes such as “respect” or “protection,” which in fact position older adults as passive, frail, and dependent. While such approaches appear to elevate older people, they simultaneously undermine their autonomy and restrict their social roles [29]. In Türkiye, widely emphasized cultural stereotypes such as “respect for elders” and “wisdom” may appear as positive values but, in practice, can lead to exclusion from decision-making processes or exposure to overprotective behaviors. Accordingly, the weaker and more heterogeneous reliability of the positive subscale in our study may be understood as a cultural reflection of this concept. On the one hand, traditional norms ascribe a positive status to older adults; on the other, everyday practices involving condescension, underestimation, or excessive paternalism reinforce a contradictory pattern between positive and negative perceptions [29,30]. Supporting this, studies conducted in Türkiye have shown that older individuals are simultaneously evaluated with both positive stereotypes, such as “wisdom” and “being worthy of respect,” and negative stereotypes such as “incompetence” and “frailty” [31]. Furthermore, the Turkish adaptation of the Ageism Survey reported that 82.5% of participants experienced at least one form of ageism, underscoring the widespread nature of ageism in Türkiye [32].

Demographic factors also provide an important perspective through which to interpret these findings. Individuals with higher education may be more critical of negative stereotypes, whereas older adults in rural areas may be more likely to emphasize traditional respect norms. In addition, given the strong family-based support systems in Turkey, older adults living with family may report fewer experiences of negative ageism, whereas those living alone may express higher levels of perceived discrimination [8]. These observations highlight how cultural norms and demographic variables may shape the structural properties of the PAQ-TR subscales.

Several limitations of this study should also be acknowledged. Because the study was conducted using a cross-sectional design, the sensitivity of the instrument to change over time could not be evaluated. The study sample was drawn from a single geographical region, and most participants had primary-level education, which may limit the generalizability of the findings. Although participants were included based on their ability to independently complete the questionnaire, no formal cognitive screening was administered, which should be considered a limitation. Data collection through self-report may also introduce social desirability. Possible clustering of responses at the extreme categories of the 5-point Likert scale was not formally examined in this study, which represents a potential limitation and should be addressed in future research with larger and more heterogeneous samples. Nevertheless, the relatively large and diverse sample may mitigate some of these limitations.

Future research should focus on applying the instrument across different age groups, clinical populations, and sociocultural contexts to strengthen generalizability. Longitudinal studies are also needed to examine the responsiveness and sensitivity of the PAQ-TR to changes over time. To enhance the reliability of the Positive subscale, the development of additional items or revisions of existing ones would be beneficial. Measurement invariance testing across gender, education, and age groups would provide further evidence of the scale’s structural robustness. Although both EFA and CFA demonstrated satisfactory model fit and met conventional psychometric criteria, future studies are recommended to enhance model robustness by conducting EFA and CFA on independent subsamples (e.g., using random or stratified splitting) or by applying cross-validation and multi-group invariance testing across larger and more diverse samples. Multi-group CFA for gender and education was considered to assess measurement invariance; however, subgroup sample sizes were insufficient for stable estimation. Future studies with larger and more balanced samples should examine configural, metric, and scalar invariance of the PAQ-TR. Finally, mixed-method studies, incorporating qualitative approaches, could yield deeper insights into how older adults perceive ageism within their specific cultural context. Also, future research should incorporate information on pre-retirement occupation, as prior professional experiences may shape older adults’ perceptions of ageism and provide additional context for interpreting PAQ-TR scores. Beyond these methodological directions, the present findings also offer guidance for future cultural adaptations of the PAQ-8. When translating the instrument into other languages, researchers should consider how local cultural values—such as respect, familial obligation, or intergenerational hierarchy—shape perceptions of aging. What appears as a positive social attitude in one culture may carry paternalistic or exclusionary meanings in another, illustrating the conceptual complexity inherent in measuring ageism across cultures. Therefore, future adaptations should evaluate not only linguistic accuracy but also the deeper cultural meanings that influence how ageism is expressed and experienced.

## 5. Conclusions

The present study demonstrated that the PAQ-TR has acceptable psychometric properties in terms of structural validity, convergent validity, and reliability. The two-factor structure (positive and negative perceptions of ageism) was supported, and test–retest analyses confirmed good temporal stability. While the negative subscale showed satisfactory internal consistency, the positive subscale revealed lower reliability, likely due to the limited number of items and cultural nuances in the perception of positive aspects of aging. These findings highlight the importance of further refinement of the positive dimension to strengthen the instrument’s psychometric robustness.

## Figures and Tables

**Table 1:** Descriptive Characteristics of Participants.

Variable	Male (n = 137)	Female (n = 170)	Total (n = 307)
**Age (years)**	71.03 (6.79)	71.33 (7.12)	71.19 (6.96)
**Height (cm)**	172.45 (7.77)	160.62 (6.78)	165.92 (9.33)
**Weight (kg)**	77.32 (12.89)	73.21 (16.11)	75.05 (14.87)
**BMI (kg/m^2^)**	26.04 (4.10)	28.43 (6.12)	27.36 (5.43)
**Dominant side**			
Right	126 (41.0)	150 (48.9)	276 (89.9)
Left	11 (3.6)	20 (6.5)	31 (10.1)
**Employment**			
Yes	3 (1.0)	1 (0.3)	4 (1.3)
No	134 (43.6)	169 (55.0)	303 (98.7)
**Education level**			
Primary school	86 (28.0)	140 (45.6)	226 (73.6)
Middle school	29 (9.4)	26 (8.5)	55 (17.9)
High school	13 (4.2)	4 (1.3)	17 (5.5)
Bachelor’s	8 (2.6)	0 (0.0)	8 (2.6)
Master’s or higher	1 (0.3)	0 (0.0)	1 (0.3)
**Chronic disease**			
Yes	58 (19.0)	122 (39.9)	180 (58.8)
No	79 (25.8)	47 (15.4)	126 (41.2)
**Medication**			
Yes	58 (19.0)	121 (39.5)	179 (58.5)
No	79 (25.8)	48 (15.7)	127 (41.5)

Values are presented as mean (standard deviation) for continuous variables and n (%) for categorical variables; BMI = Body Mass Index; n = count.

**Table 2:** Descriptive Statistics of PAQ-TR and B-APQ Scales.

Variable	Mean (SD)	Min–Max (Range)
**PAQ-TR (n = 307)**		
Positive	10.66 (2.62)	3–15 (12)
Negative	10.48 (3.87)	5–22 (17)
Total	21.14 (3.64)	12–31 (19)
**B-APQ (n = 307)**		
Timeline–chronic	10.92 (3.45)	3–15 (12)
Positive consequences	11.73 (2.72)	3–15 (12)
Positive control	10.72 (3.11)	3–15 (12)
Negative consequences/control *	16.92 (4.91)	5–25 (20)
Emotional representations	6.89 (1.89)	2–10 (8)
Total B-APQ score	57.16 (9.03)	25–79 (54)

PAQ-TR = Turkish Version of Perceived Ageism Questionnaire; B-APQ = Brief Aging Perceptions Questionnaire. SD = Standard Deviation; Min = Minimum; Max = Maximum; * Higher scores reflect more negative perceptions (reverse-coded items).

**Table 3:** Item- and scale-level content validity indices (I-CVI and S-CVI) for PAQ-TR.

	Relevance	Clarity	Comprehensiveness	Mean	κ*
**I-CVI**					
**PAQ1**	0.90	0.90	0.80	0.87	0.86
**PAQ2**	1.00	0.90	0.90	0.93	0.92
**PAQ3**	1.00	0.90	0.90	0.93	0.92
**PAQ4**	0.90	0.80	0.80	0.83	0.82
**PAQ5**	1.00	1.00	0.90	0.97	0.97
**PAQ6**	0.90	0.80	0.80	0.83	0.82
**PAQ7**	0.90	0.90	0.90	0.90	0.89
**PAQ8**	1.00	1.00	1.00	1.00	1.00
**S-CVI**	0.95	0.90	0.86	0.93	0.90

I-CVI: Item-level Content Validity Index; S-CVI: Scale-Level Content Validity Index; κ*: modified Kappa; PAQn = Item of Perceived Ageism Questionnaire.

**Table 4:** Fit indices for the two-factor CFA model of the PAQ-TR.

Model	χ^2^ (df, *p*)	χ^2^/df	CFI	TLI	RMSEA (90% CI)	SRMR	PCLOSE	HTMT
**Two** **factor (PAQPos–PAQNeg)**	23.920 (13, 0.03)	1.84	0.97	0.94	0.05 (0.01–0.08)	0.03	0.41	0.76

PAQPos = Positive Ageism; PAQNeg = Negative Ageism; χ^2^ = chi-square; df = degrees of freedom; CFI = Comparative Fit Index; TLI = Tucker–Lewis Index; RMSEA = Root Mean Square Error of Approximation; SRMR = Standardized Root Mean Square Residual; PCLOSE = test of close fit; HTMT = Heterotrait–Monotrait Ratio.

**Table 5:** Standardized factor loadings and explained variances for the items of the PAQ-TR.

Factor	Item	Loading (β)	Explained Variance (%)
**Negative Ageism**	PAQ1 (n = 307)	0.39	15.0%
	PAQ2 (n = 307)	0.57	32.7%
	PAQ4 (n = 307)	0.67	45.2%
	PAQ6 (n = 307)	0.66	43.4%
	PAQ7 (n = 307)	0.56	31.6%
**Positive Ageism**	PAQ3 (n = 307)	0.58	33.6%
	PAQ5 (n = 307)	0.43	18.6%
	PAQ8 (n = 307)	0.45	20.5%

PAQn = Item of Perceived Ageism Questionnaire.

**Table 6:** Convergent validity: correlations between PAQ-TR and B-APQ subscales.

PAQ-TR Subscale	B-APQ Domain	r	95% CI	*p*	q (FDR)
**Negative Ageism**	Timeline–Chronic	0.15	[0.04, 0.26]	0.007 **	0.012 **
	Positive Consequences	−0.29	[−0.38, −0.18]	<0.001 ***	<0.001 ***
	Positive Control	−0.23	[−0.33, −0.12]	<0.001 ***	<0.001 ***
	Negative Conseq./Control	0.31	[0.21, 0.41]	<0.001 ***	<0.001 ***
	Emotional Presentations	0.25	[0.14, 0.36]	<0.001 ***	<0.001 ***
	B-APQ Total	0.13	[0.01, 0.23]	0.028 **	0.04 **
**Positive Ageism**	Timeline–Chronic	0.001	[−0.11, 0.12]	0.944	0.950
	Positive Consequences	0.27	[0.16, 0.37]	<0.001 ***	<0.001 ***
	Positive Control	0.20	[0.09, 0.31]	<0.001 ***	<0.001 ***
	Negative Conseq./Control	−0.15	[−0.25, −0.03]	0.011 **	0.018 **
	Emotional Presentations	−0.12	[−0.23, −0.01]	0.040 **	0.045 **
	B-APQ Total	0.02	[−0.09, 0.14]	0.688	0.720

r = Spearman’s rho correlation; 95% CI = 95% confidence intervals; ** = *p* < 0.05; *** = *p* < 0.001; q (FDR) = False Discovery Rate–adjusted *p* value (Benjamini–Hochberg correction); PAQ = Perceived Ageism Questionnaire; B-APQ = Brief Aging Perceptions Questionnaire; Conseq. = Consequences.

**Table 7:** Internal consistency indices for PAQ-TR.

Subscale	Corrected Item–Total Correlation	MIIC	α if Item Deleted	α (Total)	ω
**Positive Ageism**					
**PAQ3**	0.317	0.26 (range 0.24–0.28)	0.433	0.52	0.48
**PAQ5**	0.329		0.414		
**PAQ8**	0.343		0.392		
**Negative Ageism**					
**PAQ1**	0.396	0.35 (range 0.21–0.47)	0.704	0.72	0.71
**PAQ2**	0.486		0.669		
**PAQ4**	0.538		0.647		
**PAQ6**	0.514		0.657		
**PAQ7**	0.458		0.680		

MIIC = mean inter-item correlation; α = Cronbach’s alpha; ω = McDonald’s omega; PAQn = Item of Perceived Ageism Questionnaire.

**Table 8:** Item- and subscale-based test–retest reliability of PAQ-TR.

Item	ICC	95% CI	SD (T1) (n = 307)	SD (T2) (n = 74)	SD Pooled	SEM	SDC_95_
**PAQ1**	0.82	[0.78, 0.85]	1.13	1.19	1.16	0.49	1.36
**PAQ2**	0.77	[0.72, 0.81]	1.15	1.17	1.16	0.56	1.55
**PAQ3**	0.74	[0.68, 0.79]	1.14	1.12	1.13	0.58	1.61
**PAQ4**	0.75	[0.69, 0.80]	1.14	1.10	1.12	0.56	1.56
**PAQ5**	0.72	[0.66, 0.77]	1.20	1.23	1.22	0.64	1.78
**PAQ6**	0.71	[0.64, 0.76]	1.14	1.12	1.13	0.61	1.70
**PAQ7**	0.68	[0.61, 0.74]	1.06	1.08	1.07	0.60	1.68
**PAQ8**	0.77	[0.72, 0.82]	1.31	1.30	1.31	0.62	1.72
**Positive Ageism**	0.80	[0.75, 0.83]	2.55	2.58	2.56	1.16	3.22
**Negative Ageism**	0.86	[0.83, 0.89]	3.82	3.95	3.88	1.44	3.99

ICC = Intraclass correlation coefficient; SD = Standard Deviation; SEM = Standard error of measurement; SDC_95_: Smallest Detectable Change at %95 confidence level; PAQn = Item of Perceived Ageism Questionnaire.

## Data Availability

The data and code that support the findings of this study are available from the corresponding author, Ö.E.D., upon reasonable request. Due to ethical restrictions and participant confidentiality, the dataset is not publicly available.

## Appendix A

### Appendix A.2. Item Equivalence Matrix of PAQ-TR

Item	Original English Item (Brinkhof et al., 2022)	Final Turkish Item (PAQ-TR, Appendix A1)	Semantic Equivalence	Idiomatic Equivalence	Conceptual Equivalence	Comment
1	*…people approach you as if you are less capable because of your age?*	*... ne sıklıkta size çocuk gibi yaklaştığı hissine kapıldınız?*	Equivalent	Equivalent	Equivalent	Declarative form converted to interrogative for linguistic naturalness.
2	.. you are not being listened to and/or your opinion advice is not taken seriously because of your age?	*... ne sıklıkta sizi dinlemediği ve/veya fikir önerinizi ciddiye almadığı hissine kapıldınız?*	Equivalent	Equivalent	Equivalent	Same meaning; grammatical adaptation only.
3	…people value your advice and contribution to a conversation because of your life experience?	*... ne sıklıkta tavsiyelerinize ve bir konuşmaya katkılarınıza değer verdiği hissine kapıldınız?*	Equivalent	Minor change	Equivalent	Reworded for experiential phrasing; concept retained.
4	.. people hold negative prejudices or exaggerated stereotypes (e.g., weak, vulnerable, dull, slow) about you because of your age?	*...ne sıklıkta sizin hakkınızda olumsuz önyargılarının* *veya abartılı kalıp yargılarının (örn. zayıf, savunmasız, donuk, yavaş) olduğu hissine kapıldınız?*	Equivalent	Equivalent	Equivalent	Direct translation in interrogative structure.
5	.. people assume that you are wise and sensible because of your age?	*... ne sıklıkta bilge ve mantıklı olduğunuzu varsaydığı hissine kapıldınız?*	Equivalent	Equivalent	Equivalent	Lexical harmonization; concept unchanged.
6	.. people unjustly treat you as if you are less capable (mentally or physically) because of your age?	*... ne sıklıkta size daha az becerikliymişsiniz (mental* *veya fiziksel olarak) gibi haksız yere davranış gösterdiği hissine kapıldınız?*	Equivalent	Minor change	Equivalent	“Disparagingly” rendered as “aşağılayıcı biçimde” for idiomatic accuracy.
7	.. people think disparagingly (with little or no dignity) about your contribution to society because of your age?	*... ne sıklıkta topluma katkınız ile ilgili aşağılayıcı (düşük itibar veya itibarsız) bir düşünce taşıdığı hissine kapıldınız?*	Equivalent	Minor change	Equivalent	Lexical substitution “sensible → mantıklı.”
8	.. people see you as a meaningful part of society precisely because of your age (e.g., babysitting (grand)children or volunteer activities)?	*... ne sıklıkta sizi toplumun tam olarak anlamlı bir parçası* *olarak gördüğü hissine kapıldınız? (örn. Bebek veya* *çocuk (torun) bakıcılığı ya da g.nüllü faaliyetler (örn.* *birilerine destek olmak amacıyla para kazanmadan yaptığınız temizlik, tamirat ve tadilat işleri))*	Equivalent	Minor change	Equivalent	Expanded examples to reflect culturally typical unpaid support roles.

All items were reformulated into a uniform interrogative structure beginning with *“ne sıklıkta … hissine kapıldınız mı?”* (“how often … have you had the feeling that …”) to ensure linguistic coherence and cultural naturalness while preserving conceptual fidelity. Minor lexical and idiomatic adjustments were made for clarity and cultural relevance (Items 3, 6, 7, 8).
